# Gene Therapy in Rare Genetic Disorders: Current Progress and Future Perspectives

**DOI:** 10.2174/0113892029361490250310041259

**Published:** 2025-03-13

**Authors:** Sundus Khawaja, Raja Hussain Ali, Ishtiaq Ahmed, Muhammad Umair

**Affiliations:** 1 Department of Biotechnology, University of Azad Jammu and Kashmir, Muzaffarabad, Pakistan;; 2 Department of Hematology/Oncology, Boston Children's Hospital, Harvard Medical School, Teaching Hospital, Boston, Massachusetts, USA;; 3 Medical Genomics Research Department, King Abdullah International Medical Research Center (KAIMRC), King Saud Bin Abdulaziz University for Health Sciences, Ministry of National Guard Health Affairs (MNGH), Riyadh, Saudi Arabia;; 4 Institute of Biochemistry and Biotechnology, Pir Mehr Ali Shah Arid Agriculture University Rawalpindi 46000, Pakistan;; 5 Metropole Laboratories (Private) Limited, Islamabad 44000, Pakistan

**Keywords:** Gene therapy, rare genetic disorders, *Zolgensma*, *in vivo* gene therapy, *ex vivo* gene therapy

## Abstract

Rare genetic disorders collectively affect millions of individuals worldwide, presenting a significant clinical and research challenge due to the diversity and complexity of the underlying mutations. Current treatment options are often limited, focusing on symptom management rather than addressing the root genetic causes. This review article aims to provide a perspective on the evolving field of gene therapy for rare genetic disorders, emphasizing recent advancements, current challenges, and future directions. A comprehensive review of recent advancements in gene therapy for rare genetic disorders was conducted, focusing on therapeutic strategies, delivery systems, and clinical outcomes. Key examples, such as the use of viral vectors and gene-editing technologies (*e.g*., CRISPR), were highlighted. The challenges, including immune responses and ethical concerns, were also examined. Gene therapy has achieved significant milestones, with the successful development of therapies like *Zolgensma* for spinal muscular atrophy and *Luxturna* for retinal dystrophy. However, several hurdles, including efficient gene delivery, immune reactions, and long-term safety, remain unresolved. Gene therapy holds transformative potential for the treatment of rare genetic disorders. While recent successes mark a new era in genetic medicine, ongoing research is required to refine delivery mechanisms, overcome immune-related barriers, and ensure ethical and safe therapeutic interventions.

## 1 INTRODUCTION

Rare genetic disorders, often defined as conditions affecting fewer than 1 in 2,000 individuals, represent a vast array of over 7,000 distinct diseases. These disorders affect approximately 300 million individuals globally, with the majority manifesting early in life and often leading to significant health complications, developmental challenges, and, in some cases, early mortality [1]. Rare genetic disorders are often caused by mutations in specific genes, which result in abnormal protein production, loss of function, or other cellular dysfunctions. These disorders encompass a vast range of conditions, including spinal muscular atrophy (SMA), cystic fibrosis, and hemophilia, each typically resulting from a mutation in a single gene. Because of their rarity and genetic diversity, diagnosing and treating these disorders poses significant challenges [2].

Traditional therapeutic approaches, such as symptomatic treatments and enzyme replacement therapies (ERT), have been beneficial for certain conditions. ERT, for example, has shown success in treating lysosomal storage disorders like Gaucher disease and Fabry disease by replacing the deficient enzyme [3]. Similarly, oral medicines are prescribed in case of sickle cell anemia hydroxyurea which reduces the pain and prevents dactylitis in children. However, these treatments are not curative and often require lifelong administration, which can limit their effectiveness. Furthermore, they do not address the underlying genetic cause of the disorder [4].

## 2 PREVALENCE AND TREATMENT GAPS

Because each rare genetic disorder affects a limited number of people, developing effective treatments is challenging. Traditional drug development focuses on common diseases with large patient populations, leaving rare diseases with minimal therapeutic options. As a result, most rare genetic disorders lack curative treatments, and the available therapies often only address symptoms rather than underlying causes. This lack of curative options contributes to high morbidity and mortality rates, creating a substantial unmet need for targeted therapies [5].

## 3 THE PROMISE OF GENE THERAPY

Gene therapy offers a transformative approach to treating rare genetic disorders by addressing the root cause: the genetic mutation itself. By introducing, modifying, or repairing defective genes within a patient’s cells, gene therapy has the potential to restore normal function at a molecular level. This precision can be particularly effective for rare genetic disorders, which often stem from single-gene mutations, making them ideal candidates for gene correction. Gene therapy has already led to breakthroughs in conditions like SMA and certain inherited retinal diseases, offering hope for future advances across a broader range of rare disorders [6].

Gene therapy operates on the principle of targeting and modifying genes to correct underlying genetic mutations, aiming to treat or even cure diseases at their molecular source. By directly altering DNA within cells, gene therapy can restore or replace faulty genes responsible for causing specific conditions. This approach is particularly promising for rare genetic disorders, as many of these conditions are monogenic, meaning mutations in a single gene cause them. This simplicity enables precise targeting, which increases the likelihood of effective and sustained outcomes. Unlike traditional therapies that manage symptoms, gene therapy has the potential to address the root cause of rare disorders, offering long-term relief and potentially eliminating the need for lifelong treatment [7, 8].

This review will examine the progress made in applying gene therapy to treat rare genetic disorders, highlighting key advances and successful case studies. It will also assess the current challenges facing gene therapy, including technical, regulatory, and ethical obstacles, as well as the limitations in delivery and long-term safety. Finally, the review will provide perspectives on future directions, including emerging technologies and innovative strategies, to address existing gaps and expand the potential of gene therapy as a transformative approach for treating rare genetic conditions.

## 4 GENE THERAPY: A REVOLUTIONARY APPROACH

Gene therapy offers a transformative potential for treating rare genetic disorders by directly targeting the root cause of disease at the genetic level. This approach involves the introduction, modification, or silencing of genes within a patient’s cells to correct or compensate for the genetic defect. There are two primary types of gene therapy approaches:

### 4.1 *In Vivo* Gene Therapy

In this approach, the gene-editing tools (*e.g*., CRISPR, viral vectors) are delivered directly into the patient’s body, targeting specific tissues or cells. This method is particularly useful for organs that are hard to access or for disorders affecting tissues that require localized gene correction. Direct *in vivo* gene therapy has been used in Spinal Muscular Atrophy (SMA) treatment, where the SMN1 gene is delivered using an AAV9 vector, targeting motor neurons directly. Another example includes gene therapy for retinal disorders, where viral vectors are injected into the eye to correct genetic mutations affecting vision [9, 10]. Recent advancements in the field have brought several groundbreaking therapies to the market. For example, *Zolgensma*, a gene therapy approved for spinal muscular atrophy (SMA), delivers a functional copy of the SMN1 gene *via* an adeno-associated virus (AAV) vector, restoring the production of survival motor neuron protein. Similarly, *Luxturna* was approved for Leber congenital amaurosis, a rare inherited retinal disease, and is designed to replace a defective RPE65 gene. These therapies have demonstrated remarkable success, offering not only the potential to halt disease progression but, in some cases, to reverse damage [11, 12].

### 4.2 *Ex Vivo* Gene Therapy

In this method, patient cells are extracted, genetically modified in the laboratory to introduce therapeutic genes, and then reintroduced into the patient. *Ex vivo* therapy provides better control over gene editing, allowing thorough quality control of modified cells before reintroduction. *Ex vivo* gene therapy is widely applied in hematological disorders such as sickle cell disease and beta-thalassemia, where hematopoietic stem cells are extracted, edited to correct the defective gene, and then reinfused into the patient to repopulate the blood with corrected cells [13].

Gene therapy can further be categorized based on the type of cells where genetic material is altered.

Somatic cell gene therapy involves alteration in the genetic material of somatic cells. The effect of alteration is confined to the individual and cannot be transferred to its progeny. Germline gene therapy, on the other hand, involves gene alteration of gametes. This type of therapy is heritable but is often prohibited in some countries [14].

## 5 CRISPR-BASED THERAPIES

**Fig. (1):**
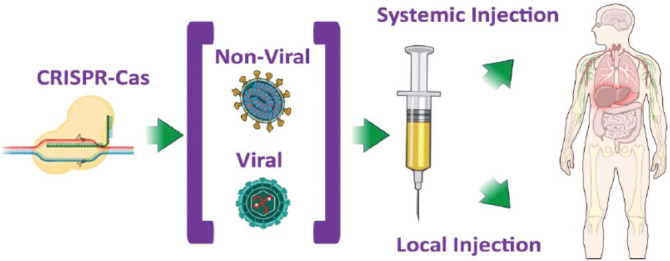
CRISPR/Cas9-based gene therapy.

CRISPR-Cas9 and related systems allow for targeted DNA editing with high precision and efficiency. The Cas9 protein, guided by a short RNA sequence, cuts the DNA at a specific site, enabling gene insertion, correction, or disruption. The discovery of CRISPR-Cas9 has brought a major breakthrough in the field of medicine by enabling the modification of DNA for gene therapy. Variations like prime and base editing have allowed scientists to correct point mutations that cause genetic disorders without increasing the off-target effects. Successful clinical trials have been launched using CRISPR for the treatment of sickle cell disease and beta-thalassemia. The aim is to activate the fetal hemoglobin for all cases of hemoglobinopathies [15, 16]. The first trial of in vivo gene editing using CRISPR was carried out in a patient with Leber congenital amaurosis (LCA), which is a rare genetic disorder affecting the eye (Fig. **1**). CRISPR is more efficient and less costly compared to earlier methods. It is also adaptable to multiplex editing, where multiple genes can be edited simultaneously, which is valuable for polygenic disorders [17].

## 6 ZINC-FINGER NUCLEASES (ZFNS) AND TALENS

These are custom-engineered proteins that bind and cut specific DNA sequences. ZFNs were the first tools used in clinical trials for gene editing, particularly in HIV resistance therapies, by disrupting the CCR5 gene in T cells. TALENs (Transcription Activator-Like Effector Nucleases) function similarly to ZFNs but allow for easier targeting and customization. TALENs have been used in experimental therapies, such as treating leukemia by targeting specific genes in T cells. While both ZFNs and TALENs offer high specificity, they are more complex and less versatile than CRISPR systems, which are more widely adopted due to CRISPR’s simpler design and ease of programming [18, 19].

## 7 BASE AND PRIME EDITING

This method enables precise point mutations without introducing double-strand breaks (DSBs), making it safer for single-nucleotide polymorphisms (SNPs) associated with genetic disorders. Cytidine deaminase or adenosine deaminase enzymes are fused to Cas proteins to alter specific DNA bases (A to G or C to T). A newer approach can perform targeted insertions, deletions, and all 12 types of base substitutions. Prime editing combines Cas9 nickase with a reverse transcriptase, allowing for complex edits with reduced off-target effects. Both base and prime editing are promising for disorders caused by single-point mutations, like cystic fibrosis, sickle cell disease, and Tay-Sachs disease. They offer increased precision, particularly useful for treating diseases with known, specific mutations [20, 21].

## 8 GENE DELIVERY VEHICLES

### 8.1 Viral Vectors

Viruses are obligate parasites infecting the human and meanwhile delivering the genome into host cells. This mechanism has made viruses a good vector for gene therapy and development of vaccine. Four major categories of viruses that have been engineered and currently in use are retroviruses (RVs), lentiviruses (LVs), adeno (Advs) and adeno associated viruses (AAVs) [22].

### 8.2 Retroviruses

**Fig. (2):**
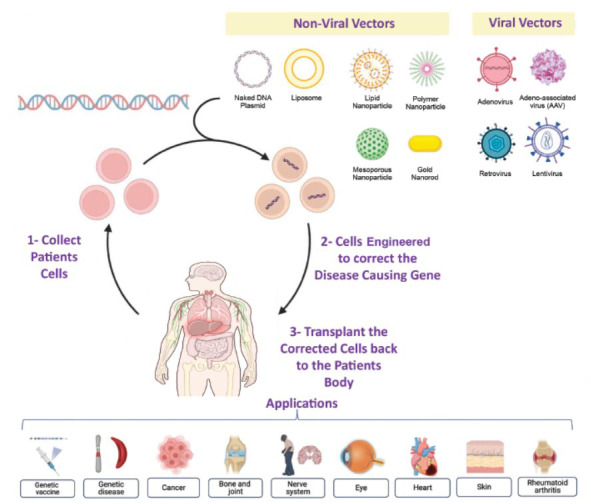
Different methods of viral delivery systems.

These are RNA viruses that are known to cause tumors in rodents. Once inside the host cell, RNA is reverse-transcribed to cDNA, and invertase inserts the cDNA into the host genome. Due to this property, they provide a sustainable delivery mode. Retroviruses have been exploited for the treatment of SCID, but the major concern is insertional mutagenesis which can lead to the development of leukemia-like symptoms (Fig. **2**) [23].

### 8.3 Lentiviruses

They are from the sub-group of retroviruses and possess 2 copies of the RNA genome. LVs can transfer the gene into both dividing and nondividing cells, so this is an efficient vector to be used for stable and sustainable targeting of broad cell types like neurons, liver, and immune cells [24]. After integration, the therapeutic gene shows expression for a long period of time. Lentiviruses have shown promising results in cases of Parkinson’s disease and spinal muscular atrophy.

### 8.4 Adenoviruses

These are double-stranded DNA viruses having a genome size of 36kb and are known for causing respiratory, intestinal and eye infections in humans. They are not integrating means the genome is only delivered into the host nucleus. They are considered ideal for short-term expression. AdV can elicit strong immune responses that may lead to vector clearance. To overcome these limitations, helper-dependent Adenoviral vectors are also known. As gutless vectors are developed that have most of the genomes removed and only retain the inverted terminal repeats and packaging system that are essential for DNA replication and encapsulation. Hence, a large amount of transgene can be incorporated and transported. AdV can deliver CRISPR/ Cas9 system and has been rated as a novel therapeutic delivery strategy [25].

### 8.5 Adeno-associated Viruses (AAV) in Gene Therapy

Adeno-associated viruses (AAV) are small, single-stranded DNA viruses that naturally integrate genetic material into a specific site on human chromosome 19, making them highly suitable for targeted gene therapy. These vectors offer lasting gene expression, although occasionally uncontrolled, which has proven beneficial for conditions like hemophilia. In hemophilia, AAV can be injected into muscle tissue to produce Factor IX, effectively reducing bleeding episodes. Notably, AAV-based therapies, such as Luxturna (using AAV2 for RPE65 mutations causing Leber congenital amaurosis) and Zolgensma (using AAV9 for spinal muscular atrophy by replacing the SMN1 gene), have shown remarkable therapeutic outcomes [26].

### 8.6 Non-viral Vectors in Gene Delivery

Non-viral vectors provide a safer alternative to viral vectors, especially for short-term expression or in cases where immune response must be minimized. These methods deliver DNA into cells using physical techniques rather than viruses.

### 8.7 Liposomes

Liposomes are lipid-based vesicles that can encapsulate therapeutic DNA, allowing it to merge directly with cell membranes to release genetic material into target cells. They are highly versatile, biocompatible, and often have lower immunogenicity than viral vectors, making them safer for repeated administrations. Liposomes can be chemically modified to improve specificity for certain tissues and protect DNA from degradation, which has been particularly useful for targeted cancer therapies and vaccines [27].

### 8.8 Beta-cyclodextrin Based Nanoparticles

Advancements in non-viral gene delivery, particularly through cell-penetrating nanoparticles like β-cyclodextrin (β-CD), show promise as safer alternatives to viral vectors. These nanoparticles are made up of cyclic oligosaccharides that form a hydrophobic cavity capable of encapsulating therapeutic molecules, making them ideal for both drug and gene delivery. Their non-toxic nature enhances specificity, drug solubility, and stability. β-CD nanoparticles were first used in a clinical trial in 2010 to treat metastatic melanoma patients by delivering siRNA intravenously to downregulate RRM2 mRNA [28]. These nanoparticles have also shown promise in delivering an anti-cancer drug, Paclitaxel, and a siRNA to downregulate the toxic Huntington protein (HTT) in Huntington's disease [29, 30]. Due to their stable release properties, β-CD nanoparticles are now being investigated as a potential treatment for Wolfram Syndrome. Their ability to deliver therapeutic genes safely and effectively makes them a promising avenue for curing this rare, monogenic, neurodegenerative disease, which affects nearly 30,000 people worldwide [31, 32].

Wolfram Syndrome (WFS) is a rare, neurodegenerative disorder caused by mutations in the WFS1 or CISD2 genes, leading to severe multi-organ dysfunction, including diabetes, optic atrophy, and hearing loss. This condition has no cure, and existing treatments are largely ineffective in improving life expectancy or quality of life. Current research is focused on understanding the genetic and molecular mechanisms underlying WFS1, particularly the consequences of WFS1 gene deletion, which causes profound endoplasmic reticulum (ER) stress and proteostasis disturbances. Given the lack of effective treatment, innovative strategies, such as gene therapy, are being explored to address the fundamental cause of the disease [28, 29].

### 8.9 Magnetofection

Magnetofection combines magnetic particles with DNA, allowing the DNA-magnetic complex to be directed into target cells using an external magnetic field. This method enhances transfection efficiency, especially for hard-to-transfect cells such as neurons and stem cells. Due to its precision and high local concentration, magnetofection has potential applications in targeted therapies, particularly for diseases requiring localized gene expression, like in neurodegenerative disorders [33].

### 8.10 Electroporation

Electroporation uses high-voltage electrical pulses to create temporary pores in cell membranes, enabling DNA to enter. Although highly effective, electroporation can lead to cell damage due to the electric field intensity. To minimize cell loss, optimized protocols and low-voltage variants are being developed, broadening their use in gene therapy for genetic diseases, immunotherapy, and tissue engineering [34].

### 8.11 Gene Gun/Biolistics

The gene gun, or biolistic delivery, propels DNA-coated gold or tungsten particles directly into cells by high-velocity helium bursts. This approach bypasses cellular uptake limitations and is suitable for tissue-targeted therapies, particularly in areas like skin and ocular applications where localized transfection is necessary. Despite its benefits, gene gun techniques are primarily limited to external or accessible tissues due to the need for direct physical contact with the tissue [35].

## 9 TRANSCRIPTOMICS: A GUIDE TO GENE THERAPY

Recent advancements in genomic technologies, particularly in transcriptomics, have significantly enhanced our ability to understand dynamic changes in DNA and RNA over time and in response to various challenges. Transcriptomics, which involves the study of all RNA present in cells, plays a critical role in understanding cell phenotypes and diseases [36]. Despite much of the transcriptome not translating into proteins, it still impacts cellular function and provides insight into complex pathologies. This growing field has expanded the understanding of gene functions, particularly by challenging traditional views such as the definition of “pseudogenes,” which have been found to be transcribed and even translated. RNA sequencing, a powerful tool in transcriptomics, has proven essential for analyzing genetic, neurodegenerative, and cancer-related diseases by providing comprehensive sequence data, revealing alternative splicing mechanisms, and identifying key transcriptional regulators [37]. Integrating transcriptomic data with genomic information can refine gene therapy approaches, ensuring that treatments are tailored to the genetic complexities of diseases at the cellular level, ultimately improving the precision and efficacy of therapies. Moreover, transcriptomics aids in evaluating the effectiveness of gene delivery vectors, a critical component for successful gene transfer. As such, transcriptomics is not only instrumental in disease diagnosis and clinical trial design but also serves as a valuable tool in the development of personalized gene therapies, advancing the field of precision medicine [38].

## 10 SUCCESS STORIES IN GENE THERAPY FOR RARE DISEASES

### 10.1 Zolgensma (Onasemnogene Abeparvovec) for Spinal Muscular Atrophy (SMA)

SMA is a severe neurodegenerative disorder caused by mutations in the *SMN1* gene, leading to motor neuron loss and muscle atrophy. Zolgensma, developed by Novartis, is a gene therapy that delivers a functional copy of the *SMN1* gene *via* an adeno-associated virus (AAV9) vector directly to motor neurons [39].

Zolgensma was approved by the U.S. FDA in 2019 for the treatment of pediatric patients under 2 years of age with SMA. Clinical trials have demonstrated that patients treated with Zolgensma showed significant improvement in motor function and survival compared to untreated patients, offering the potential for long-term benefits. Zolgensma has dramatically changed the treatment landscape for SMA, providing a one-time infusion that can halt disease progression, potentially saving lives and preventing severe disability [40].

### 10.2 Luxturna (Voretigene Neparvovec) for Inherited Retinal Disease

Inherited Retinal Diseases, such as Leber Congenital Amaurosis (LCA), is caused by mutations in the RPE65 gene, leading to progressive vision loss. Luxturna, developed by Spark Therapeutics, is a gene therapy that introduces a functional copy of the RPE65 gene into retinal cells using an AAV2 vector [41].

Luxturna was approved by the U.S. FDA in 2017 for the treatment of adult and pediatric patients with inherited retinal disease caused by biallelic RPE65 mutations. In clinical trials, patients who received Luxturna demonstrated significant improvements in visual function, such as an increased ability to navigate in low-light conditions, which was sustained over time. Luxturna marked a major milestone as the first FDA-approved gene therapy for a genetic retinal disease, offering the potential to restore vision and improve the quality of life for patients with previously untreatable forms of inherited blindness [42].

These case studies exemplify the success of gene therapy in providing lasting, potentially curative treatments for rare genetic disorders. They highlight how advancements in gene delivery, vector development, and patient selection are transforming the treatment landscape, offering new hope to patients with previously incurable conditions.

### 10.3 On-going Clinical Trails

Clinical trials in gene therapy for rare disorders have made significant strides, especially in conditions like cystic fibrosis, muscular dystrophy, and hemophilia.

### 10.4 Cystic Fibrosis (CF)

CF, caused by mutations in the CFTR gene, has seen advances through clinical trials using viral and non-viral vectors to deliver functional copies of the CFTR gene to lung cells. Although challenges remain in achieving efficient gene delivery to lung tissue, ongoing trials have shown improved respiratory function in some patients and continue to refine methods for safer and more effective delivery systems [43].

### 10.5 Muscular Dystrophy

In particular, Duchenne muscular dystrophy (DMD) has been a major focus for gene therapy. Trials using adeno-associated virus (AAV) vectors to deliver a shortened but functional version of the dystrophin gene have shown promising results, with treated patients experiencing improved muscle strength and reduced muscle damage. This approach, while still under evaluation, offers hope for a therapy that could slow or halt disease progression [44].

### 10.6 Hemophilia

Hemophilia A and B, caused by deficiencies in clotting factors VIII and IX, respectively, have shown remarkable progress in gene therapy trials. AAV vectors delivering functional copies of the missing clotting factor genes have been able to sustain normal or near-normal clotting factor levels in patients for extended periods. These promising outcomes have led to breakthroughs, with some therapies receiving regulatory approval, offering patients the potential for long-lasting, if not lifelong, relief from frequent clotting issues [45, 46].

Overall, these clinical trials underscore the potential of gene therapy to not only treat but potentially cure certain rare genetic disorders by addressing the root cause of each condition. They also highlight ongoing efforts to improve vector safety, dosing protocols, and delivery efficiency to achieve more robust, durable results across various patient populations [47].

## 11 CHALLENGES IN GENE THERAPY FOR RARE GENETIC DISORDERS

Despite the promise, several hurdles remain in advancing gene therapy for rare genetic disorders. One significant challenge is the immune response to viral vectors, Particularly AAVs which can limit the effectiveness and safety of the treatment. Additionally, delivering gene therapy to target tissues, such as crossing the blood-brain barrier for neurological disorders, remains a complex obstacle [48].

Long-term efficacy is another concern. The longevity of the therapeutic effect, as well as potential adverse effects from gene integration into the genome, needs further investigation through long-term clinical follow-ups. To overcome these barriers, research is focused on improving vector design, utilizing non-viral delivery systems, and advancing CRISPR-based gene-editing technologies, which offer greater precision and reduced risk of off-target effects [49]. Gene augmentation, which involves introducing functional copies of defective genes, has seen significant success, particularly in monogenic disorders such as spinal muscular atrophy (SMA). CRISPR-Cas9, a precise gene-editing tool, has been instrumental in correcting mutations responsible for conditions like Duchenne Muscular Dystrophy (DMD) [32].

These non-viral methods offer advantages in transient gene expression, ideal for conditions needing temporary gene expression or frequent redosing. However, improving delivery efficiency and minimizing side effects remain key challenges. Current research focuses on enhancing target specificity, optimizing delivery mechanisms, and developing hybrid systems that combine non-viral and viral delivery benefits. By improving these aspects, non-viral vectors are set to play an increasingly significant role in safe and personalized gene therapies [27] provided the vectors address the significant bottleneck of precise tissue targeting [50, 51].

## 12 BIOLOGICAL AND TECHNICAL BARRIERS-IMMUNE REACTIONS

Most gene therapy techniques rely on viral vectors (*e.g*., adeno-associated viruses (AAV), lentivirus) to deliver therapeutic genes into cells. However, the body’s immune system may recognize these vectors as foreign invaders, leading to an immune response that can neutralize the viral vectors before they deliver their genetic payload. This immune reaction may also lead to inflammation or tissue damage, limiting the effectiveness of the therapy [22].

In some cases, individuals may already have pre-existing immunity to certain viral vectors, particularly AAVs, due to prior natural infections or vaccinations. This can significantly reduce the efficacy of the gene therapy, as the immune system rapidly clears the vectors before they can be effective. To mitigate these risks, researchers are exploring novel, less immunogenic viral vectors and immune suppression strategies to enhance vector persistence [50].

## 13 DELIVERY TO TARGET TISSUES (CROSSING THE BLOOD-BRAIN BARRIER)

One of the main challenges in gene therapy is the ability to deliver the therapeutic gene to the specific tissues or cells that require treatment. For example, in diseases like cystic fibrosis, the therapeutic gene must be delivered specifically to the lung cells, and in muscular dystrophy, the muscle cells. The size and type of vector used in gene therapy may limit its ability to reach target tissues [51] efficiently. For instance, AAV vectors can be too large to penetrate certain tissues or organs effectively, particularly in the case of tissues like the brain or lungs. Additionally, ensuring that the gene is delivered to the correct cells within the target tissue (*e.g*., motor neurons, liver cells) remains a significant challenge. While *ex vivo* gene therapy (where cells are modified outside the body and then reintroduced) offers more control over the cell delivery process, *in vivo* therapy (direct delivery to the patient’s body) faces more challenges in achieving targeted and precise gene delivery [52].

## 14 LIMITATIONS IN GENE-EDITING PRECISION- OFF-TARGET EFFECTS

One of the primary concerns in gene editing techniques, such as CRISPR-Cas9, is the potential for off-target mutations. These unintended changes to the genome could lead to harmful consequences, including disruption of other essential genes or activation of oncogenes, which could potentially lead to cancer. While technologies like CRISPR are highly precise, they may not always make the desired edit in every cell, leading to incomplete or inconsistent therapeutic outcomes. Achieving 100% editing efficiency in all target cells remains a significant challenge [53].

## 15 ETHICAL CONSIDERATIONS

One of the most significant ethical concerns in gene therapy, particularly with CRISPR and other genome-editing technologies, is germline editing—modifying the genetic material in embryos or reproductive cells. Germline modifications could be passed on to future generations, raising concerns about unintended consequences, such as the introduction of harmful mutations and the potential for “designer babies.” Another ethical issue is the potential for gene therapy to exacerbate existing health disparities. High treatment costs and limited availability of these therapies could mean that only wealthier populations have access, leaving others without a life-saving treatment. The ethical implications of gene therapy also extend to the unknown long-term effects. While short-term clinical trials may show promise, there is still uncertainty about how these therapies will perform over decades, especially when the treatments involve irreversible genetic modifications [54].

## 16 REGULATORY LANDSCAPE

The approval of gene therapies is a complex and lengthy process that involves extensive clinical trials to demonstrate safety and efficacy. Regulatory bodies like the U.S. FDA and the European Medicines Agency (EMA) are still developing frameworks to handle gene therapies, which do not always fit within the traditional drug approval process. The lack of clear and efficient pathways for approval can delay access to promising therapies. Gene therapies often involve permanent changes to a patient's genetic material, necessitating long-term post-treatment monitoring to assess potential delayed side effects, such as the development of cancer or immune-related issues. Establishing adequate regulatory protocols for such monitoring remains an area of active discussion and development.

## 17 HIGH COST, COMPLEX DEVELOPMENTAL PROCEDURES, ETHICAL CONCERNS, AND LIMITED PATIENT’S AVAILABILITY

**Fig. (3):**
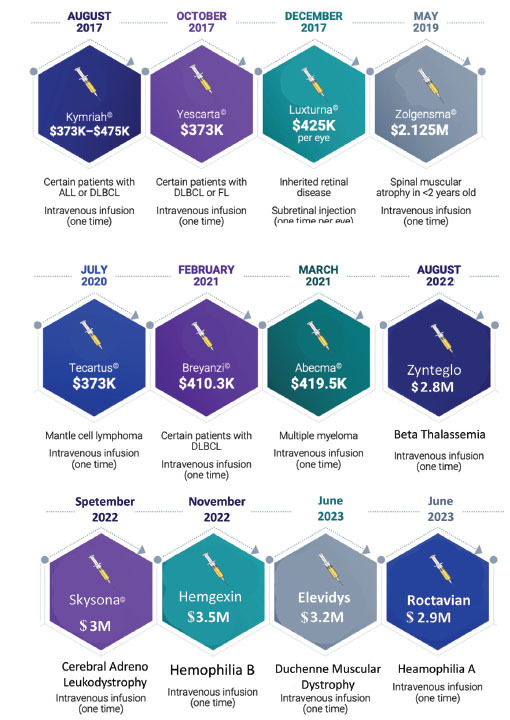
FDA-approved gene therapies and their cost.

Gene therapy's high cost stems from multiple factors related to research, development, production, and clinical application. Developing gene therapies requires years of research and clinical trials involving advanced technologies, high-skilled personnel, and large investments. Only a fraction of therapies make it through trials, adding to the cost of successful treatments. Hemgenix, a gene therapy for hemophilia B, is one of the most expensive treatments globally, costing around $3.5 million per dose. This high price reflects its one-time administration potential to reduce the need for lifelong factor replacement therapy. Zolgensma, designed for Spinal Muscular Atrophy, costs around $2.1 million for a single-dose treatment. It targets the genetic cause of SMA, aiming to replace the missing or defective SMN1 gene essential for motor neuron health. Manufacturing viral vectors or non-viral delivery systems is intricate and requires specialized facilities, rigorous safety protocols, and quality control. Unlike small-molecule drugs, gene therapies are highly customized, meaning each production cycle is unique and costly (Fig. **3**).

Approval processes for gene therapies are stringent and resource-intensive, with high compliance standards. Post-approval, administering these therapies demands skilled professionals and often specialized healthcare settings. Many gene therapies target rare genetic disorders, meaning fewer patients contribute to covering the costs, unlike drugs for widespread conditions.

## 18 INSERTIONAL MUTAGENESIS

Many gene therapies use viral vectors to deliver therapeutic genes into cells. However, these vectors can integrate their genetic material into the host genome, potentially disrupting normal cellular processes. Insertional mutagenesis occurs when this integration happens near an oncogene (a gene that can cause cancer), potentially leading to tumorigenesis. This was a significant concern in earlier gene therapy trials, particularly in hematopoietic stem cell gene therapies, where unintended integration caused leukemia in some patients [27]. Newer vector designs and gene-editing technologies aim to minimize the risk of insertional mutagenesis by targeting safer, less gene-dense regions of the genome or using non-integrating vectors, but this remains a critical area of research. While gene therapies may show promising results in the short term, the long-term effects of permanent genetic alterations are still largely unknown. For example, changes to the immune system, off-target mutations, or the persistence of therapeutic genes could have long-term consequences. There is a need for continuous monitoring and long-term follow-up in clinical trials to fully understand the durability of the treatment and the potential risks associated with these permanent changes [55].

## 19 FUTURE DIRECTIONS

### 19.1 Analyzing Population-based Genetic Diversity

The future of gene therapy relies heavily on expanding our understanding of genetic diseases through studies that incorporate a diverse range of populations. Historically, genetic studies have focused primarily on single ancestral populations, limiting the scope of our knowledge. However, recent genome-wide multi-ancestral studies have significantly broadened this understanding [56, 57]. For example, a multi-ancestry genome-wide study has uncovered multiple target genes for early prediction of systemic lupus erythematosus. The inclusivity of diverse ancestries in genetic research allows for the identification of a broader spectrum of genetic variants, which can inform more effective, personalized treatment strategies [56]. Similarly, another large-scale multi-ancestry meta-analysis of Parkinson’s disease (PD), involving over 49,000 PD cases and more than 2.4 million controls from various populations (European, East Asian, Latin American, and African), identified 78 genome-wide significant loci, including 12 potentially novel ones. These findings provide valuable insights into PD's genetic basis across different ethnic groups and pave the way for future research in non-European populations [57]. Furthermore, the data generated from these studies should be shared globally to enhance gene therapy development, ultimately benefiting global health. In cancer genomics, the creation of standardized DNA reference datasets has also been crucial. Reference call sets from paired tumor-normal genomic DNA samples from a breast cancer cell line have helped identify somatic mutations and germline variants with high confidence despite not being directly representative of primary cancer cells. These reference samples play a key role in minimizing biases in sequencing technologies and assays, and they serve as a valuable resource for improving tumor genomics analyses. Together, these population-based reference samples and multi-ancestry studies contribute significantly to advancing gene therapy and precision medicine, ensuring treatments are tailored to the genetic diversity of global populations [56, 57].

### 19.2 Advances in CRISPR/Cas9 and Genome Editing

The CRISPR/Cas9 system has revolutionized the field of genome editing, allowing for precise and efficient modification of the genome. Advances in CRISPR technology, such as base editing and prime editing, are improving precision and reducing the risks of off-target effects [58].

### 19.3 Development of Non-Viral Delivery Systems

Non-viral delivery systems, such as nanoparticles, are gaining attention as safer alternatives to viral vectors. These systems could potentially reduce immune reactions and enhance the targeted delivery of therapeutic genes [59].

### 19.4 Personalized Gene Therapies

The future of gene therapy lies in personalized approaches, where treatments are tailored to the individual’s genetic makeup. Advances in next-generation sequencing and personalized medicine are paving the way for customized gene therapies [58].

## CONCLUSION

Gene therapy has emerged as one of the most promising approaches for treating rare genetic disorders, offering the potential for long-term and, in some cases, curative solutions. Over the past decade, significant progress has been made, exemplified by the success of therapies like Zolgensma, Luxturna, and hemophilia B treatments. These advancements underscore the potential of both *in vivo* and *ex vivo* gene therapies in addressing previously untreatable conditions [60]. Gene therapy has proven to be a transformative approach for treating rare genetic disorders, offering hope where none previously existed. By directly targeting the underlying genetic mutations, gene therapy addresses the root cause of these diseases, providing the potential for long-term, even permanent, cures. Successful therapies such as Zolgensma for Spinal Muscular Atrophy (SMA) and Luxturna for inherited retinal diseases have already demonstrated the remarkable ability of gene therapy to reverse disease progression, improve quality of life, and reduce dependency on lifelong treatments. These breakthroughs mark a significant turning point in medicine, not only for rare diseases but also for the broader field of genetic medicine [61].

However, challenges such as immune responses, delivery limitations (*e.g*., crossing the blood-brain barrier), and ethical concerns, particularly around genome editing, remain significant obstacles that the field must overcome. As research continues, breakthroughs in CRISPR-based genome editing, non-viral delivery systems, and the development of personalized gene therapies are likely to drive the next wave of innovations [62, 63].

Looking ahead, it is crucial to focus on refining these therapeutic strategies, expanding accessibility, and addressing the ethical and regulatory challenges that accompany gene therapy's rapid growth. With continued advancements, gene therapy holds the promise of transforming the landscape of rare genetic disorder treatment, offering hope to patients and families affected by these conditions. Despite the promising advances, there remains much to be done to overcome the challenges associated with gene therapy [64]. Continued research is essential to refine delivery methods, improve precision in gene editing, and develop safer, more effective treatment protocols. Enhanced regulatory frameworks will be crucial to ensure that gene therapies are safely approved, while also addressing long-term monitoring and accessibility issues. Furthermore, innovation in gene-editing technologies and alternative delivery systems will be key to making these therapies more efficient, cost-effective, and available to a wider range of patients. As these advancements unfold, the ultimate goal is to make gene therapy a viable, accessible treatment option for the millions of individuals affected by rare genetic disorders around the world, ensuring that its benefits reach the broadest patient populations possible.

## LIST OF ABBREVIATIONS

AAV: Adeno-associated Virus
DMD: Duchenne Muscular Dystrophy
DSBs: Double-strand Breaks
EMA: European Medicines Agency
ER: Endoplasmic Reticulum
ERT: Enzyme Replacement Therapies
HTT: Huntington Protein
LCA: Leber Congenital Amaurosis
PD: Parkinson’s Disease
SMA: Spinal Muscular Atrophy
SNPs: Single-nucleotide Polymorphisms
TALENs: Transcription Activator-Like Effector Nucleases
WFS: Wolfram Syndrome
β-CD: β-cyclodextrin
